# SEM1 Downregulates HSPA8 to Suppress TLR4/MyD88/NF-κB Signaling and Alleviate Myocardial I/R Injury

**DOI:** 10.3390/ijms27156711

**Published:** 2026-07-27

**Authors:** Jingjing Liu, Jia Kang, Zhanghui Guan, Dong Tian, Yuyan Huang, Xiao Tang, Xinping Chen

**Affiliations:** 1 School of Basic Medical Sciences, University of South China, Hengyang 421001, China; 19892823496@163.com (Y.H.); tang_xiao0710@163.com (X.T.); 2 Department of Pharmacy, Lanzhou University, Lanzhou 730000, China; kangj2021@lzu.edu.cn (J.K.); guanzhh2023@lzu.edu.cn (Z.G.); tdong2023@lzu.edu.cn (D.T.); 3 Southeast Research Institute, Lanzhou University, Putian 351152, China; 4 State Key Laboratory of Applied Organic Chemistry, Lanzhou University, Lanzhou 730000, China

**Keywords:** SEM1, HSPA8, myocardial I/R injury, TLR4/MyD88/NF-κB

## Abstract

Inflammation plays a pivotal role in the pathogenesis of myocardial ischemia/reperfusion (I/R) injury, highlighting inflammation suppression as a critical therapeutic strategy. The inflammatory response is largely mediated through Toll-like receptor 4 (TLR4), a transmembrane signal receptor whose expression is upregulated by Heat Shock Protein Family A Member 8 (HSPA8). Here, we investigated whether SEM1, a subunit of the 26S proteasome, interacts with HSPA8 and attenuates TLR4-mediated inflammation. Our results demonstrate that SEM1 expression is downregulated following myocardial I/R. Overexpression of SEM1 alleviated cardiac injury and dysfunction, inhibited myocardial inflammation, and downregulated HSPA8 expression in the I/R-injured heart. Co-IP assays confirmed a strong physical interaction between SEM1 and HSPA8, while the precise molecular mechanism responsible for SEM1-induced downregulation of HSPA8 remains to be fully elucidated. Mechanistically, SEM1 suppressed the activation of the TLR4/MyD88/NF-κB signaling pathway. Collectively, these findings identify SEM1 as a novel regulator that protects against myocardial I/R injury via its association with HSPA8 and inhibition of the TLR4-mediated inflammatory cascade, offering a promising therapeutic target for this condition.

## 1. Introduction

Cardiovascular disease (CVD) remains the leading cause of mortality worldwide, accounting for more than 17.3 million deaths annually [1]. Among the spectrum of cardiovascular disorders, myocardial I/R injury represents a major challenging clinical condition, imposing a substantial economic and social burden [2]. The injury occurs when coronary blood flow is transported back to the cardiac muscle following an acute myocardial infarction, most times through revascularization operations such as PCI [3,4] or thrombolytic therapy. Paradoxically, although reperfusion is applied for rescuing ischemic myocardium, restored blood flow can itself exacerbate cellular damage, a symptom called myocardial I/R injury. This reperfusion-induced damage significantly compromises the benefits of revascularization and brings many side effects.

The underlying pathophysiology of myocardial I/R injury is highly complicated and multifactorial. Key mechanisms include an abrupt burst of reactive oxygen species (ROS) upon reperfusion [5,6], overloaded intracellular calcium [3,7], activation of a robust inflammatory response [3,8], and destructive mitochondrial dysfunction [3,9,10]. These processes collectively contribute to cardiomyocyte death, microvascular dysfunction, and irreversible tissue damage [3,11]. Consequently, there is a pressing and continuous need to investigate the intricate mechanisms driving I/R injury and to develop novel, optimal therapeutic strategies aimed at mitigating its detrimental effects and improving prognosis in patients.

Building upon the complex pathophysiology of myocardial I/R injury, particularly the roles of oxidative stress and inflammatory activation, recent research has begun to identify specific molecular players that modulate these detrimental processes. Among them, SEM1, also known as DSS1, has emerged as a protein of interest. SEM1 was originally identified for its role in human genetics, where its disruption is associated with split hand/split foot malformation (SHFM) [12]. Beyond its developmental role, SEM1 is known to participate in a multitude of critical biological functions and cellular processes [13]. A potential connection between SEM1 and I/R injury lies in the ubiquitin–proteasome system, because the system targets and degrades oxidized proteins [14,15]. Given the explosive production of ROS during reperfusion, SEM1 may help the brain to counter the damage derived from ROS.

The connection to cardiovascular injury is further highlighted through an interaction with HSPA8. HSPA8 itself has been identified as one of the potential candidates for forming UVB-induced adducts with SEM1 [14]. More pertinently, HSPA8 has been demonstrated to exacerbate post-ischemic myocardial inflammation and cardiac dysfunction by stimulating the NF-κB pathway [16]. Corroborating this, independent studies have shown that inhibition of HSPA8 attenuates spinal cord I/R injury via the astrocytic NF-κB/NLRP3 inflammasome pathway [17]. Based on these converging lines of evidence, we hypothesized that SEM1 participates in the pathogenesis of cardiovascular diseases, specifically myocardial I/R injury, through its interaction with HSPA8.

However, the precise role of SEM1 in the context of myocardial I/R remains unexplored. It is unclear whether SEM1 modulates the injury response and, if so, by what mechanism. Therefore, this study was designed to directly investigate the impact of SEM1 on cardiac function, myocardial infarct size, and the inflammatory response in a rat model of myocardial I/R injury. Furthermore, we aimed to explore the potential molecular mechanism behind this regulatory effect, with particular attention to the modulation of HSPA8 protein levels induced by SEM1 overexpression.

## 2. Result

### 2.1. SEM1 Alleviates Cardiac Injury and Dysfunction in Myocardial I/R Injury

**Figure 1:**
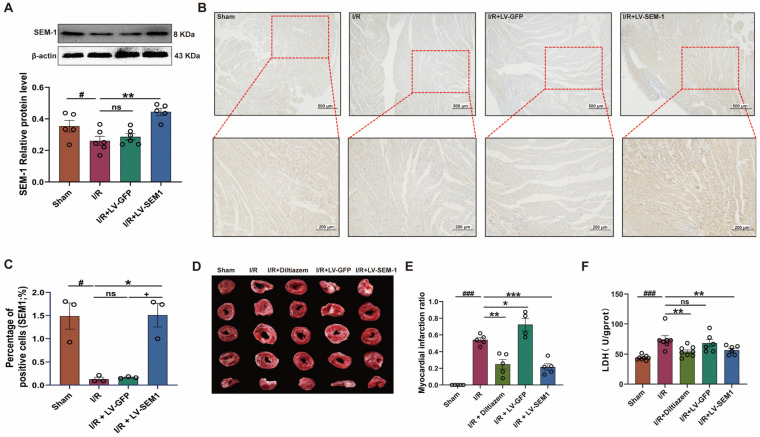
SEM1 alleviates cardiac injury and dysfunction in a rat model of myocardial I/R injury. (**A**) Western blot analysis of SEM1 protein expression levels in heart tissue from Sham and I/R groups. SEM1 expression was significantly decreased following I/R injury. (**B**,**C**) IHC analysis and semi-quantitative statistical results of SEM1 expression in myocardial tissue. Representative IHC images show SEM1-positive cells (brown staining). (**D**,**E**) Assessment of myocardial infarction area. Representative images of heart sections stained with TTC. Viable tissue is stained red, while the infarct area appears pale. (**F**) Measurement of LDH activity in heart tissues. Elevated LDH activity, an indicator of cardiomyocyte damage, was observed in the I/R group. SEM1 treatment significantly attenuated the I/R-induced increase in LDH release (*p* < 0.01 vs. I/R group). All data are represented as mean ± SEM of at least six rats in each group in vivo, ns > 0.05, ^#^ *p* < 0.05, ^###^ *p* < 0.001 as compared to Sham group, * *p* < 0.05, ** *p* < 0.01 and *** *p* < 0.001 as compared to I/R group, ^+^ *p* < 0.05, as compared to I/R + LV-GFP group.

We chose SD rats to create the model of myocardial I/R injury by ligating the anterior descending branch of the left coronary artery and then releasing the ligation to restore the blood flow [18]. Here, the ischemia lasted for 45 min while the reperfusion persisted for 24 h. Firstly, to investigate the expression changes of SEM1 in the myocardial I/R injury model, we performed immunohistochemistry and Western blot analysis on cardiac tissues. The results showed that the expression of SEM1 was markedly lowered in myocardial I/R injury compared to the Sham group (Figure 1A, *p* < 0.05). Immunohistochemistry also indicated a marked decrease in SEM1 positivity in the model group (Figure 1B,C, *p* < 0.05). These findings suggest that SEM1 may be involved in regulating the pathological progression of myocardial I/R injury. Subsequently, we constructed a lentivirus expressing SEM-1 (LV-SEM1) and delivered it to rats. Following I/R injury, both Western blot and immunohistochemical analyses confirmed a significant upregulation of SEM1 protein expression, along with a markedly increased positive staining rate, in the myocardium of LV-SEM1-treated rats (Figure 1A–C, *p* < 0.01, *p* < 0.05). These results demonstrate that lentivirus-mediated delivery of the SEM1 gene successfully and efficiently induces robust overexpression of the SEM1 protein in the myocardium of rats subjected to I/R injury. This establishes a critical experimental foundation for subsequent investigations into the specific function and molecular mechanisms of SEM1 in myocardial ischemia/reperfusion injury.

**Figure 2:**
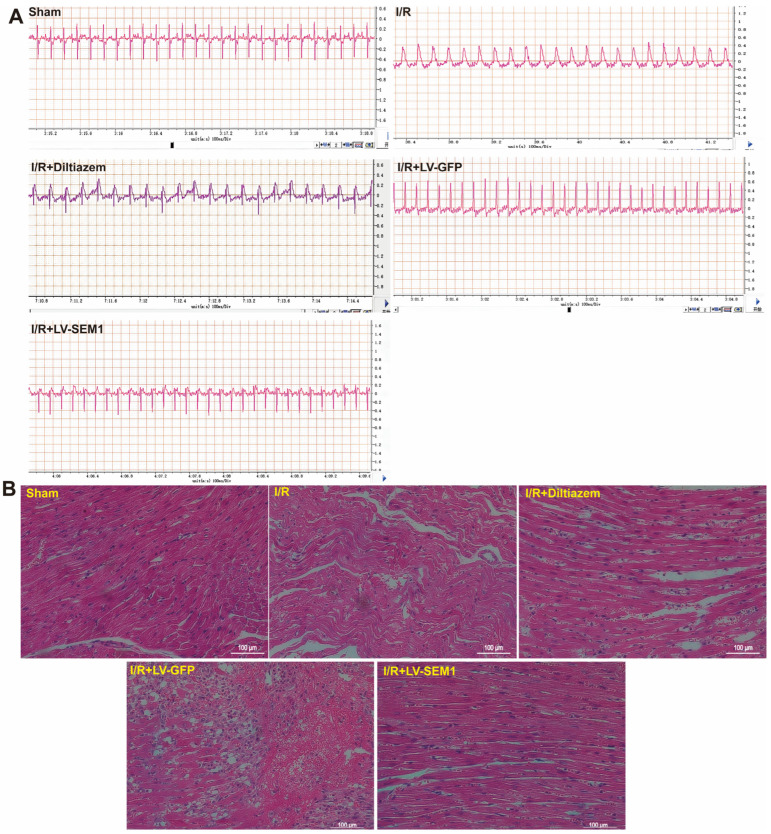
SEM1 ameliorates cardiac arrhythmias and pathological damage induced by myocardial I/R injury. (**A**) Representative electrocardiogram (ECG) recordings (sweep speed: 100 ms/division). ECG from the Sham group shows normal sinus rhythm. The I/R group exhibits severe arrhythmias and ST-segment elevation, indicative of acute myocardial ischemia and electrical instability. In contrast, the I/R + LV-SEM1 treatment group shows significant alleviation of arrhythmias and attenuation of ST-segment changes. (**B**) Representative H&E-stained histological sections. In the Sham group, cardiac muscle fibers are arranged in orderly, parallel bundles with clear cross-striations. Cardiomyocytes display normal morphology, intact membranes, and centrally located nuclei, with no evident inflammatory infiltration or interstitial edema. The I/R group shows severe structural disorganization, including widespread cardiomyocyte necrosis, marked inflammatory cell infiltration, interstitial edema, and hemorrhage. Treatment with SEM1 (I/R + LV-SEM1 group) markedly attenuated the pathological damage: myocardial architecture is better preserved, with significantly reduced necrosis, less inflammatory infiltration, and milder interstitial edema compared with the I/R group.

Next, we detected the myocardial infarct area by TTC staining and observed that LV-SEM1 relieved infarction in rats subjected to I/R injury (Figure 1D,E, *p* < 0.001). LDH is usually taken as a marker of cellular damage, and its activity indicates the degree of myocardial damage [19]. We found that LDH activity was dramatically reduced in LV-SEM1 rats (Figure 1F, *p* < 0.01). Meanwhile, the ECG analysis showed that LV-SEM1 effectively minimized the amplitude of the ST-segment elevation and lowered it to less than 0.2 mv in I/R rats (Figure 2A). To further explore the ability of SEM1 to alleviate myocardial damage, we performed a HE staining and found that myocardium in the infarction area became loose and chaotic with telangiectasia and fibroblasts in the I/R rats (Figure 2B). However, compared with the I/R rats, myocardial cells at the infarction border in the LV-SEM1 rats were arranged in an orderly fashion, with fewer fibroblasts and limited telangiectasia (Figure 2B). We also constructed LV-GFP lentivirus as the control in this animal study. According to the results (Figure 1 and Figure 2), we found no obvious improvements in the LV-GFP-treated myocardial infarction rats. Specifically, their infarct size, abnormal electrocardiogram and enhanced LDH activity remained unchanged. Therefore, simple overexpression of any irrelevant gene cannot produce cardioprotective effects. In addition, these data further prove that SEM1 specifically alleviates myocardial injury.

### 2.2. SEM1 Inhibits Inflammation of Myocardial Tissue Experiencing Myocardial I/R Injury

**Figure 3:**
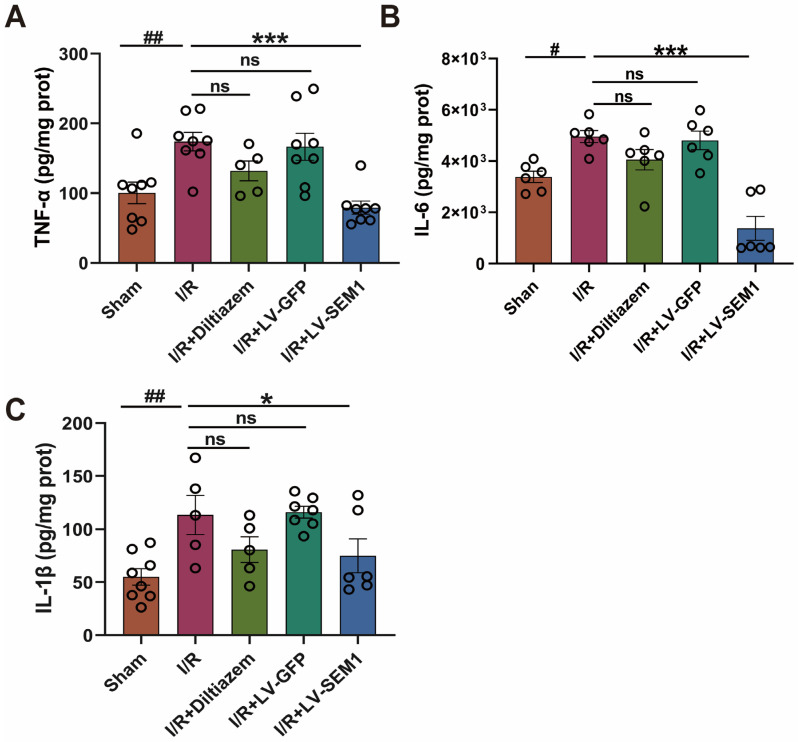
ELISA analysis of pro-inflammatory cytokines in myocardial tissue homogenates. Quantification of (**A**) TNF-α, (**B**) IL-6, and (**C**) IL-1β protein concentrations. Myocardial I/R injury significantly increased the levels of all three inflammatory cytokines compared to the Sham group. Overexpression of SEM-1 via lentiviral delivery (I/R + LV-SEM1 group) significantly reduced their concentrations compared to the I/R group. All data are represented as mean ± SEM of at least six rats in each group or at least three independent experiments in vivo, ns > 0.05, ^#^ *p* < 0.05, ^##^ *p* < 0.01 as compared to Sham group, * *p* < 0.05,and *** *p* < 0.001 as compared to I/R group.

Since inflammation plays a key role in myocardial injury [20], we next examined the extent of cardiac inflammatory responses in myocardial I/R rats. As shown in Figure 3, remarkedly elevated levels of inflammatory cytokines including TNF-α, IL-6 and IL-1β were shown in myocardial I/R rats compared with those in Sham rats (Figure 3A–C, *p* < 0.01, *p* < 0.05, *p* < 0.01). By contrast, the amounts of TNF-α, IL-6 and IL-1β were notably downregulated in myocardial tissues of LV-SEM1 rats subjected to I/R (*p* < 0.001, *p* < 0.001, *p* < 0.05). In addition, the levels of inflammatory factors also remained unaltered in the LV-GFP-treated group compared with I/R rats. These results demonstrate that SEM1 suppresses inflammatory responses in myocardial tissues after cardiac injury.

### 2.3. SEM1 Lowers HSPA8 to Prevent Myocardial I/R Injury

**Figure 4:**
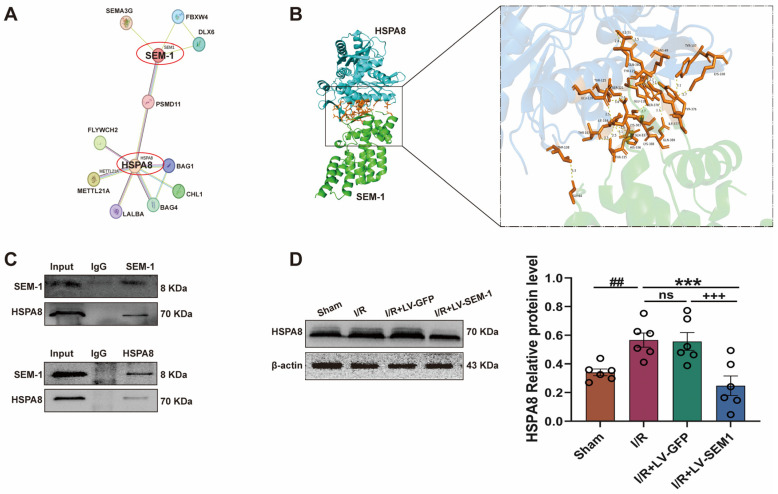
HSPA8 is identified as a downstream target of SEM-1. (**A**) Protein–protein interaction network prediction. A STRING database analysis was performed to predict potential binding partners of SEM-1. The network highlights HSPA8 as a high-confidence candidate for interaction with SEM-1. (**B**) Molecular docking simulation. Protein–protein docking of SEM1 (shown in green) and HSPA8 (shown in blue) was performed via the HDOCK platform. The optimal conformation was screened using shape complementarity and ITScorePP scoring functions. The two proteins exhibited favorable spatial interface matching with a simulated binding energy of −241.7 kcal/mol with a ligand root-mean-square deviation (L-RMSD) of 72.90 Å. (**C**) Co-IP validation of the interaction. Lysates from heart tissue were subjected to Co-IP using an anti-SEM-1 antibody or control IgG, followed by immunoblotting with anti-HSPA8 and anti-SEM-1 antibodies. HSPA8 was specifically co-precipitated with SEM-1, confirming their direct physical interaction in vivo. (**D**) Western blot analysis of HSPA8 protein expression. Protein levels of HSPA8 were analyzed in myocardial tissues from the Sham, I/R, I/R + LV-GFP and I/R + LV-SEM1 groups. I/R injury markedly elevated HSPA8 expression relative to the Sham group. Notably, SEM1 overexpression (I/R + LV-SEM1 group) significantly reversed the I/R-induced upregulation of HSPA8, whereas GFP overexpression (I/R + LV-GFP group) exerted no such effect. All data are represented as mean ± SEM of at least three independent experiments in vivo, ^##^ *p* < 0.01 as compared to Sham group, ns > 0.05, *** *p* < 0.001 as compared to I/R group, ^+++^ *p* < 0.001 as compared to I/R + LV-GFP group.

We further investigated the mechanism by which SEM1 ameliorates myocardial I/R injury in rats. Previous studies have indicated that HSPA8 plays an important role in cardiovascular diseases [16], and HSPA8 has been reported as a binding partner of SEM1 [14]. To validate the interaction between SEM1 and HSPA8, we first performed bioinformatic analysis and molecular docking studies. STRING protein interaction database analysis suggested a potential interaction between SEM-1 and HSPA8 (Figure 4A), supporting their functional association. The study conducted protein–protein molecular docking simulations of SEM1 and HSPA8 using the HDOCK platform. The optimal docking conformation was selected utilizing the shape complementarity scoring function and the ITScorePP scoring system. The simulations revealed a favorable interface matching in spatial structure between SEM1 and HSPA8, indicating the potential formation of a stable spatial docking complex. Furthermore, the binding energy analysis demonstrated a simulated binding energy of −241.7 kcal/mol for this docking model (Figure 4B). Subsequently, to verify this interaction in myocardial tissue, we conducted Co-IP assays. The results demonstrated that HSPA8 and SEM1 could physically interact in cardiac tissue (Figure 4C). Notably, overexpression of SEM1 significantly reduced the protein expression level of HSPA8 (Figure 4D, *p* < 0.001). These findings indicate that SEM1 can directly bind to HSPA8 in myocardial tissue, potentially regulating its protein stability, thereby reducing the abnormal accumulation of HSPA8 following myocardial I/R injury. This discovery links the cardioprotective effect of SEM1 to the functional regulation of HSPA8, laying an important foundation for further elucidating its downstream signaling pathways.

### 2.4. SEM1 Suppresses TLR4/MyD88/NF-κB Pathway to Improve Myocardial I/R Injury

**Figure 5:**
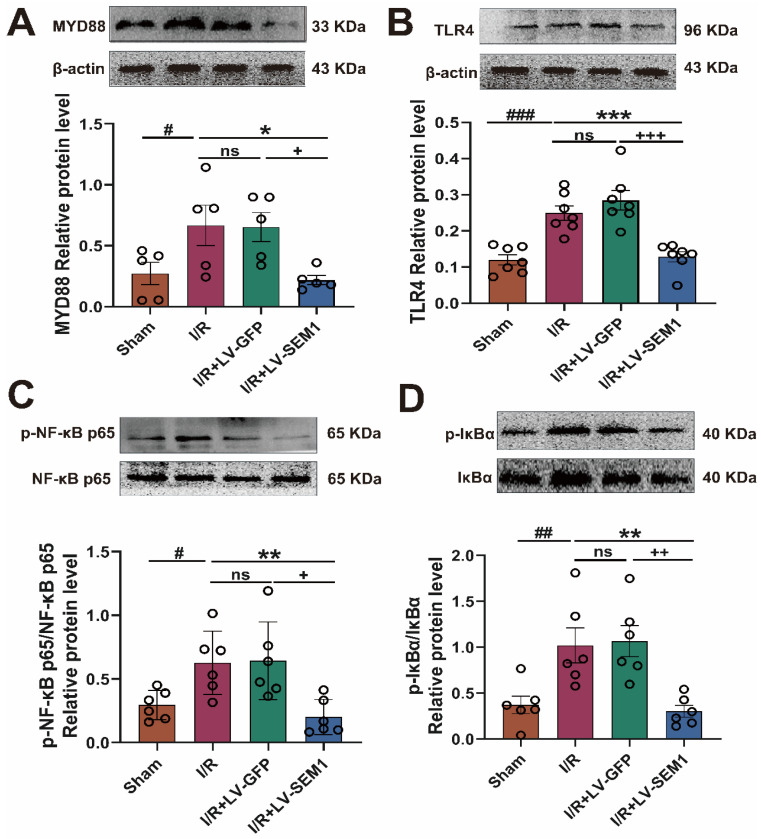
SEM1 suppresses TLR4/MyD88/NF-κB signaling in myocardial I/R injury. Relative protein levels of (**A**) MYD88, (**B**) TLR4, (**C**) p-NF-κB p65/NF-κB p65, and (**D**) p-IκBα/IκBα in myocardial tissues from the Sham, I/R, I/R + LV-GFP, and I/R + LV-SEM1 groups. I/R injury significantly upregulated MYD88 and TLR4 expression and increased the phosphorylation ratios of NF-κB p65 and IκBα. Overexpression of SEM1 effectively reversed these changes. All data are represented as mean ± SEM of at least three independent experiments in vivo, ^#^ *p* < 0.05, ^##^ *p* < 0.01 and ^###^ *p* < 0.001 as compared to Sham group; ns > 0.05, * *p* < 0.05, ** *p* < 0.01 and *** *p* < 0.001 as compared to I/R group; ^+^ *p* < 0.05, ^++^ *p* < 0.01 and ^+++^ *p* < 0.001 as compared to I/R + LV-GFP group.

Previous studies have uncovered that blockade of TLR4/MyD88/NF-κB signaling reduces myocardial inflammation, thereby improving myocardial injury in patients receiving percutaneous coronary intervention [19]. Additionally, HSPA8 induced NF-κB activation in a TLR4-dependent manner, thereby exacerbating cardiac dysfunction [16], and it is reasonable to propose that SEM1 suppresses TLR4/MyD88/NF-κB signaling. Thus, we examined the major proteins of the TLR4/MyD88/NF-κB pathway to explore how SEM1 affects inflammation as well as I/R. As expected, the amounts of MyD88, TLR4, p-NF-κB p65 and p-IκBα in I/R rats were higher than those in Sham rats (Figure 5). Instead, LV-SEM1 downregulated those proteins in the hearts of I/R rats (Figure 5). These results indicate that SEM1 can exert a suppressive effect on the cardiac inflammatory response by lowering the expression of the TLR4/MyD88/NF-κB pathway. In conclusion, our data proves that SEM1 diminishes HSPA8 amount to block TLR4-mediated inflammation and thereby alleviates myocardial ischemia and reperfusion injury.

## 3. Discussion

Myocardial I/R injury is prevalent among the population, particularly in patients experiencing acute myocardial infarction, which is a primary pathology of coronary artery disease (CAD) [20,21]. Ischemia initiates inflammatory cascade, while reperfusion amplifies this response. The sudden restoration of oxygen and blood flow enhances robust pro-inflammatory signaling pathways, thereby exacerbating tissue damage [3,8,9,22]. Much evidence has proved that this maladaptive inflammatory response causes I/R injury, supporting it as a practical therapeutic target for cardioprotection [8,22,23]. Our research provides the direct evidence supporting the notion that exogenous SEM1 offers significant protection against myocardial I/R injury by effectively suppressing the inflammatory response.

To assess the functional significance of SEM1, we conducted an experiment by intraperitoneally injecting LV-SEM1 to enhance SEM1 expression. Firstly, we determined the lentivirus administration protocol based on the published literature [24,25,26]. Lentivirus was delivered via intraperitoneal injection for 7 consecutive days, with an injection volume of 500 μL per rat. Secondly, to verify the infection efficiency of LV-SEM1 in rats, we performed in vitro cellular validation first. Fluorescence imaging was conducted on 293T cells transfected with LV-SEM1 at 24 h post transfection, which confirmed the lentiviral vector worked properly to express target genes (Supplementary Figure S1A). After that, paired bright-field and fluorescence imaging was performed on rat heart slices. The results demonstrated transgenic fluorescence signals could be detected in myocardial tissue post systemic lentiviral administration via intraperitoneal injection (Supplementary Figure S1B,C). This intervention effectively shielded the heart from I/R injury, leading to significant cardioprotective effects. These effects were characterized by a pronounced attenuation of the local inflammatory response (Figure 3) and a decrease in LDH release (Figure 1E), a crucial indicator of cellular necrosis. Consequently, these enhancements resulted in a superior functional outcome, notably reducing myocardial infarct size (Figure 1C,D).

SEM1, an essential component of the 26S proteasome responsible for degrading aberrant proteins within cells [27], plays a crucial role in combating protein damage induced by reactive oxygen species during myocardial I/R injury [28]. Despite its established function in maintaining cellular protein homeostasis, the specific involvement of SEM1 in the pathogenesis of myocardial I/R injury remains poorly understood. To address this gap in knowledge, our study aimed to comprehensively investigate the impact of SEM1 on myocardial I/R injury. Our primary and pivotal discovery revealed a significant decrease in SEM1 expression levels in the myocardial tissues of rats subjected to I/R injury compared to Sham-operated controls (Figure 1A–C). This diminished presence of SEM1 implies a potential compromise in the proteasomal system’s ability to eliminate damaged proteins under I/R conditions, potentially leading to the accumulation of harmful proteins and exacerbation of cellular damage.

We have proposed a model to explain the cardioprotective effects of SEM1 overexpression, focusing on its regulatory interaction with HSPA8. Previous research has demonstrated that HSPA8 enhances inflammation by increasing TLR4-dependent NF-κB signaling, a pathway known to exacerbate cardiac dysfunction following ischemia [16,29]. Moreover, the inflammatory upregulation induced by HSPA8 has been found to worsen spinal cord tissue damage during ischemia–reperfusion [17]. Interestingly, previous studies have hinted a potential direct interaction between SEM1 and HSPA8 [14]. This interaction was further confirmed through molecular docking experiments (Figure 4**)**, indicating a stable binding between the two proteins. This raises a puzzling question: if SEM1 and HSPA8 interact directly, how does SEM1 overexpression result in an anti-inflammatory effect? This intriguing question has prompted us to delve deeper into the functional implications of this interaction.

Our experimental data resolve this contradiction by confirming the specific Co-IP of SEM1 with HSPA8 (Figure 4C), validating their physical interaction in the complex cellular environment of the myocardium. Importantly, instead of enhancing the pro-inflammatory effects of HSPA8, our results demonstrate that this interaction promotes the downregulation of HSPA8 protein expression (Figure 4D). Thus, we conclude that SEM1’s significant therapeutic potential in myocardial injury models lies in its ability to interact with and subsequently reduce the levels of HSPA8. By binding to HSPA8, potentially through its inherent 26S proteasome activity, SEM1 effectively disrupts a crucial component of the NF-κB-mediated inflammatory pathway, thereby mitigating the adverse effects of myocardial I/R injury.

TLR4 represents a pivotal mediator of innate immunity, being the first human Toll-like receptor working as a membrane protein for pathogen recognition [30]. Once activation by its ligands, TLR4 initiates the production of pro-inflammatory cytokines and chemokines, and the process is classic inflammatory response [31]. A substantial body of evidence has firmly established the critical role of TLR4-mediated inflammation in the pathogenesis of cardiovascular diseases [32]. Specifically, genetic deficiency of *TLR4* or the application of its pharmacological antagonists has been shown to improve myocardial I/R injury by blocking the NF-κB pathway [33]. Corroborating this, studies in rat models of coronary microembolization (CME) demonstrated marked activation of the TLR4/NF-κB axis, while its inhibitor TAK-242 alleviated myocardial injury efficiently [34]. Furthermore, the cardioprotective effects of agents like nicorandil have been largely attributed to their ability to intercept the TLR4/MyD88/NF-κB signaling pathway [19]. In our current study, we observed that SEM1 overexpression potently suppresses the release of key pro-inflammatory cytokines, including TNF-α, IL-6, and IL-1β, in the context of myocardial I/R injury (Figure 3A–C). Given our parallel finding that SEM1 downregulates HSPA8, a known upstream amplifier of TLR4-mediated inflammation [35,36], we logically hypothesized that the anti-inflammatory effect of SEM1 is executed through the negative regulation of the TLR4 pathway.

**Figure 6:**
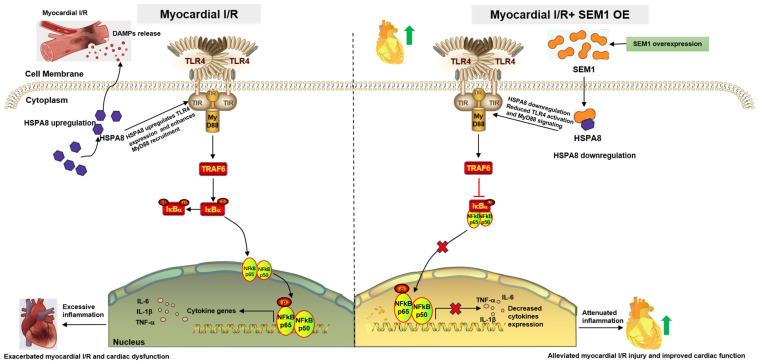
Proposed schematic model of the molecular mechanism underlying the cardioprotective effect of SEM1 overexpression in myocardial I/R injury. SEM1 modulates TLR4-mediated inflammation by downregulating HSPA8 expression, thereby inhibiting the activation of the TLR4/MyD88/NF-κB signaling pathway and attenuating myocardial I/R injury.

Supporting this hypothesis, our subsequent experimental data confirmed that treatment with LV-SEM1 significantly shut off the activation of the TLR4/MyD88/NF-κB signaling pathway in the injured myocardium (Figure 5). Therefore, the combined results from our animal model strongly suggest that the potent anti-inflammatory properties of SEM1 observed in myocardial I/R injury are mechanistically achieved, at least in part, through the suppression of the HSPA8/TLR4/MyD88/NF-κB axis. The schematic diagram of this signaling pathway is shown in Figure 6. This delineates a novel and coherent molecular pathway through which SEM1 confers cardioprotection.

Based on the comprehensive experimental evidence presented in this study, we conclude that SEM1 confers significant cardioprotection in the setting of myocardial I/R injury. The underlying mechanism is primarily mediated through its functional interaction with HSPA8. By downregulating HSPA8 expression, SEM1 effectively suppresses the subsequent activation of the TLR4/MyD88/NF-κB signaling pathway. This cascade inhibition culminates in a marked reduction in the maladaptive inflammatory response, a key driver of tissue damage in I/R [37]. These findings not only elucidate a novel molecular pathway through which SEM1 operates but also establish a foundational rationale for exploring its therapeutic potential in the treatment of cardiovascular diseases.

However, this study has limitations as the precise molecular mechanism through which SEM1 downregulates HSPA8 protein levels remains unelucidated with direct experiments. Multiple potential regulatory pathways need validation. SEM1 may hinder HSPA8 gene transcription by modulating its promoter activity and altering transcription factor binding efficiency, thereby reducing mRNA available for protein synthesis. At the translation level, SEM1 could disrupt ribosome assembly, microRNA-mediated translation inhibition pathways, or mRNA stability, thereby directly impeding HSPA8 protein synthesis efficiency. Post-translationally, abnormal protein transport, intracellular protein sorting issues, reduced molecular chaperone complex binding stability, and increased extracellular HSPA8 secretion may collectively decrease total intracellular HSPA8 protein content. Despite being a 26S proteasome subunit, the potential role of SEM1 in mediating HSPA8 degradation via the proteasome pathway is speculative without experimental support in this study, serving as a theoretical conjecture insufficient to explain HSPA8 downregulation reliably. Further research is imperative for a comprehensive understanding.

## 4. Materials and Methods

### 4.1. Reagents

Medlab (MD3000-E) was purchased from Zhenghua Biologic Apparatus Facilities (Huanbei, China), and a small animal ventilator (ZH-DW3000B) was ordered from Zhenhua Educational Equipment Co., Ltd (Henan, China). Primary antibodies including anti-NF-κB p65 (Cat# BA0610), anti-TLR4 (Cat# A00017-3), anti-HSPA8 (Cat# PB0678), and anti-MYD88 antibodies (Cat# A00025-2) were purchased from Boster Biological Technology (Beijing, China). Anti-phospho-IKBα antibody (Cat# 14D4) was ordered from Cell Signaling (Shanghai, China). Anti-DSS1 antibody (SEM-1, Cat# DF12152) was from Affinity Biosciences (Changzhou, China). Anti-β-actin antibody (Cat# GB15003) was from Servicebio (Wuhan, China). Lactate dehydrogenase assay kit (E-BC-K046-M), Rat IL-1β ELISA kit (E-EL-R0012c), Rat IL-6 ELISA kit (E-EL-R0015) and Rat TNF-α ELISA kit (E-EL-R2856c) were purchased from Elabscience Biotechnology (Wuhan, China).

### 4.2. Construction of the Recombinant Lentiviruses

The pLenti-CMV-GFP Neo (657-2) (P4870, MiaoLingPlasmid, Wuhan, China) plasmid was amplified in Escherichia coli Stbl3 cells. The SEM1 coding sequence from pET-28a-SEM1 was inserted into the pLenti-CMV construct digested with XbaI and SalI to get pLenti-CMV-SEM1. After thawing and subculturing HEK 293T cells to a density of 80%, they were put into a 10 cm cell culture plate. The plasmids (pLenti-CMV-GFP Neo (657-2)/pLenti-CMV-SEM1: pMD2.G: psPAX2 = 2:1:1) were added to the HEK 293T cells to package the lentiviruses when cells reached an 80% density. The lentiviruses were subsequently collected with a universal Virus Concentration Kit (C2901S, Beyotime Biotechnology, Shanghai, China).

### 4.3. Animal Experiment

Adult male SD rats (250 ± 20 g) were purchased from the Veterinary Research Institute of Chinese Academy of Agricultural Sciences in Lanzhou, China. The rats were housed with free access to food and water under standard conditions and preserved in 12 h light/dark as well as 45–50% humidity at 25 ± 1 °C. All animal experiments were approved by the Animal Ethics Committee of Lanzhou University (SYXK (Gan) 2018-0002).

Before surgery, the 50 animals were divided randomly into five groups.

Sham group: Surgery without I/R (*n* = 10).

I/R group: Rats were subjected to I/R surgery (*n* = 10).

I/R + Diltiazem: Diltiazem (positive control, 20 mg/kg) was administered daily by gavage for 7 days prior to I/R (*n* = 10).

I/R + LV-GFP: LV-GFP was administered daily intraperitoneally for 7 days before I/R (*n* = 10).

I/R + LV-SEM1: LV-SEM1 treatment for 7 days intraperitoneally before I/R (*n* = 10).

The SD rat myocardial I/R model was established as reported [18]. In brief, SD rats were anesthetized with phenobarbital sodium, and then intubated through the mouth with a small animal ventilator. The signals for electrocardiography (ECG) were recorded with the second lead ECG monitor, and then the chest hair was shaved, and the skin was treated with 70% ethanol before cutting along the fourth rib at the left edge of the sternum to separate the muscles and open the chest, thus exposing the heart and tearing the heart capsule open. Consequently, the left anterior descending coronary artery was exposed and ligated, with the blood vessel below the ligation site turning white. The ST-segment rise in the II-lead ECG monitoring marks the success of the model. After ligation for 45 min, reperfusion was performed for 24 h.

### 4.4. TTC Staining

After modeling, the heart samples were collected and washed with PBS 3 times. Then the samples were placed in a −80 °C fridge for 10 min, and afterwards the hearts were cut into slices with thickness of 2–3 mm each. The slices were immersed in 1.5% 2,3,5-triphenyltetrazolium chloride TTC solution at 37 °C for 45 min. Then, the slices were photographed with a digital camera, and the infarction was analyzed by ImageJ1.8.0 (National Institutes of Health, Bethesda, MD, USA).

### 4.5. Hematoxylin and Eosin (H&E) Staining

The heart samples from different groups were immediately fixed with 4% paraformaldehyde for 24 h. The samples (*n* = 3 in each group) were embedded with paraffin, cut into segments of 4 μm and then stained with H&E. The samples were imaged and evaluated with a fluorescence microscope (IX73 + DP74, Olympus, Shanghai, China).

### 4.6. Immunohistochemistry (IHC)

Immunohistochemical analysis was applied to quantify the SEM1 density. The ventricular sections were processed, followed by deparaffinization and rehydration. Thereafter, the sections were rendered for heat-mediated antigen retrieval and then incubated with 3% H_2_O_2_ to block endogenous peroxidase, followed by incubation with anti-SEM1 antibody (1:200) for 12 h at 4 °C. The sections were then incubated with horseradish peroxidase (HRP)-labeled secondary antibody for 50 min at 37 °C, followed by staining with 3,3′-diaminobenzidine (DAB). Finally, the average optical density of SEM1 was analyzed and calculated by ipwin32 IPP6.0 software (*n* = 3 in each group).

### 4.7. Docking

This study utilized protein–protein molecular docking analysis to predict the potential interaction interface between SEM1 and HSPA8. All docking simulations were conducted using the HDOCK online docking platform. The protein crystal structure of SEM1 (PDB:3T5X) and HSPA8 (PDB:4H5T) was obtained from the RCSB Protein Data Bank database as the initial input structures. Prior to docking, water molecules and excess heteroatoms were removed from the original structure, hydrogen atoms were added, and energy minimization optimization was performed to prepare the protein conformations for subsequent molecular docking simulations. A rigid-body docking mode was employed to execute global search operations. The docking conformations were thoroughly evaluated using an improved shape-based pairwise scoring function and the protein–protein dedicated scoring function ITScorePP. The binding free energy of the docking output was reported in kcal/mol. The docking conformations and the results of the protein–protein interactions were visualized and processed using PyMOL 3.0.3 software.

### 4.8. Co-Immunoprecipitation (Co-IP) Assay

Proteins (1 mg in each group) from heart tissue were obtained and immunoprecipitated correspondingly with primary antibodies against SEM1, HSPA8 and IgG for 4 h at 4 °C. Then 30 μL of protein A/G agarose beads was added. After incubation at 4 °C for 5 h, bound proteins were eluted with lysis buffer. Finally, the input and eluted proteins were detected by Western blotting.

### 4.9. Western Blotting Analysis

Proteins from heart tissues were extracted with Radio Immunoprecipitation Assay (RIPA) lysis buffer (BL504A, Biosharp, Suzhou, China) supplemented with 1% PMSF (BL507A, Biosharp, Suzhou China). The supernatant obtained post 15 min of centrifugation (13,860× *g*, 4 °C) was collected for protein analysis. For each lane, 20 μg of proteins was loaded for sodium dodecyl sulfate-polyacrylamide gel electrophoresis (SDS-PAGE), and then the proteins on the gel were transferred to polyvinylidene difluoride (PVDF) membranes (Millipore, MA, USA). The membranes were blocked with 5% skimmed milk (BD Biosciences, CA, USA) powder in PBST solution for 2 h at room temperature. After blocking, the membrane was cut into pieces based on the molecular weights of target proteins and incubated with primary antibodies against SEM1 (1:500), HSPA8 (1:500), TLR4 (1:500), MYD88 (1:500), IκBα (1:500), p-IκBα (1:500), NF-κB p65 (1:500), p-NF-κB p65 (1:500) and β-actin (1:2000), followed by incubation with a HRP-conjugated secondary antibody (1:5000) at room temperature for 1 h. Immunodetection was carried out using an ECL detection reagent (BL520A, Biosharp, Shanghai, China) and the Fluorescent & Chemiluminescence Gel Imaging System (Peiqing JS-1075, Peiqing Science and Technology Co., Ltd., Shanghai, China).

### 4.10. Statistical Analysis

All the data analyses were performed by Prism software 8.3 and IBM SPSS Statistics, version 22. The data were shown as the standard error of the mean (SEM). Statistical differences in means were assessed by using one-way analysis of variance (ANOVA) followed by LSD’s post hoc test in case of multiple conditions. *p*-values below 0.05 were considered statistically significant.

## 5. Conclusions

In conclusion, this study has thoroughly investigated a novel mechanism: SEM1 mitigates myocardial I/R injury through the regulation of inflammatory responses. For the first time, the research confirms that the 26S proteasome subunit SEM1 can functionally interact with HSPA8. By inhibiting the excessive activation of the downstream TLR4/MyD88/NF-κB inflammatory signaling pathway, SEM1 effectively reduces the myocardial infarction area in vivo and suppresses the myocardial inflammatory cascade. This intervention improves cardiac injury and functional impairments resulting from myocardial I/R. Moreover, this study establishes SEM1 as a potential therapeutic target for cardiovascular diseases.

A technical limitation of this study was the inability to establish stable SEM1-knockdown or knockout cellular models despite multiple attempts. This constraint necessitated the reliance on in vivo approaches for mechanistic validation. While the animal experiments provide compelling physiological evidence, the absence of complementary in vitro loss-of-function data limits the resolution at which cell-type-specific contributions can be delineated. Future studies utilizing conditional knockout models or cell-type-specific targeting strategies would be valuable to dissect the autonomous versus non-autonomous functions of SEM1 in myocardial protection.

## Data Availability

The data are deposited on local resources and are available upon request.

## Supplementary Materials

The following supporting information can be downloaded at https://www.mdpi.com/article/10.3390/ijms27156711/s1.
