# Supplementary material for: SEM1 Downregulates HSPA8 to Suppress TLR4/MyD88/NF-κB Signaling and Alleviate Myocardial I/R Injury
Source: Int J Mol Sci. 2026 Jul 27;27(15):6711. doi: 10.3390/ijms27156711 (PMC13466264; doi:10.3390/ijms27156711)
Supplement: Supplementary file 1 [file ijms-27-06711-s001.zip › ijms-4401863-supplementary.pdf]

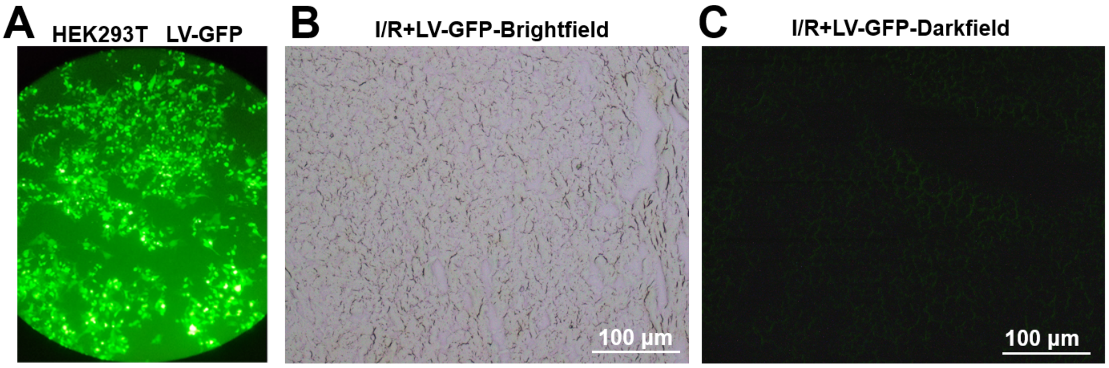

Supplementary Figure S1. (**A**) Fluorescence micrographs of HEK293T cells after transfection with LV-GFP plasmid, exhibiting obvious GFP fluorescent signals; (**B**) Brightfield images of cardiac tissue sections derived from rat injected with LV-GFP lentivirus, presenting the general histological structure of myocardium; (**C**) Fluorescence photographs of the identical LV-GFP-injected heart sections, where specific GFP fluorescent signals were readily observed.
